# Supplementary material for: Novel pelagiphage isolate Polarivirus skadi is a polar specialist that dominates SAR11-associated bacteriophage communities at high latitudes
Source: ISME J. 2023 Jul 14;17(10):1660–70. doi: 10.1038/s41396-023-01466-1 (PMC10504331; doi:10.1038/s41396-023-01466-1)
Supplement: Supplementary file 2 — Supplementary Figures [file 41396_2023_1466_MOESM2_ESM.pdf]

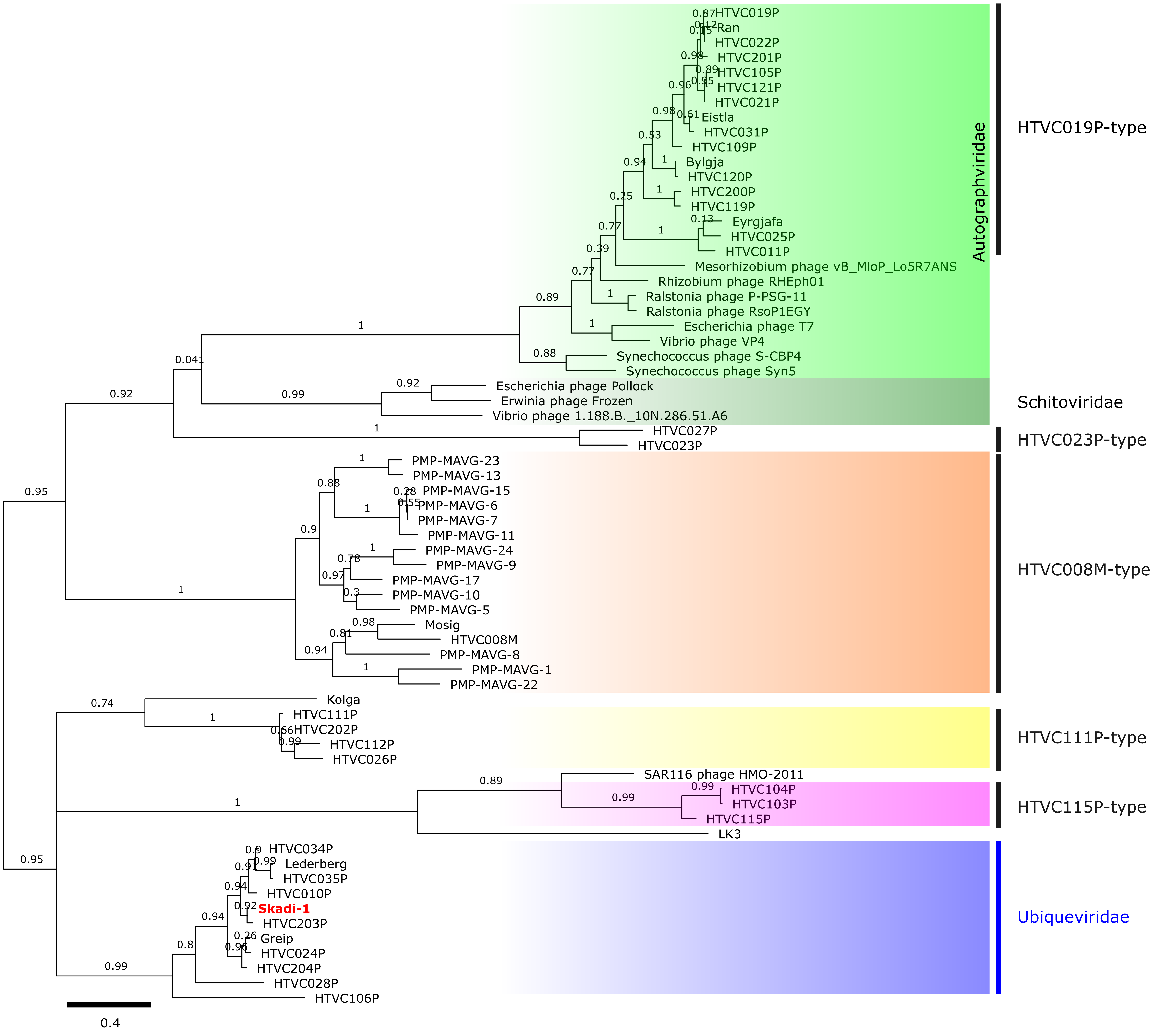

Supplementary Figure 1 Phylogeny of pelagiphage TerL genes. Unrooted neighbour-joining tree (100 bootstraps) of the TerL gene found in Pelagibacter phages and representatives of other viral families. Branches are coloured to highlight the different taxonomic groups in relation to the proposed Ubiqueviridae. Pelagibacter phage genome types are marked by black bars.

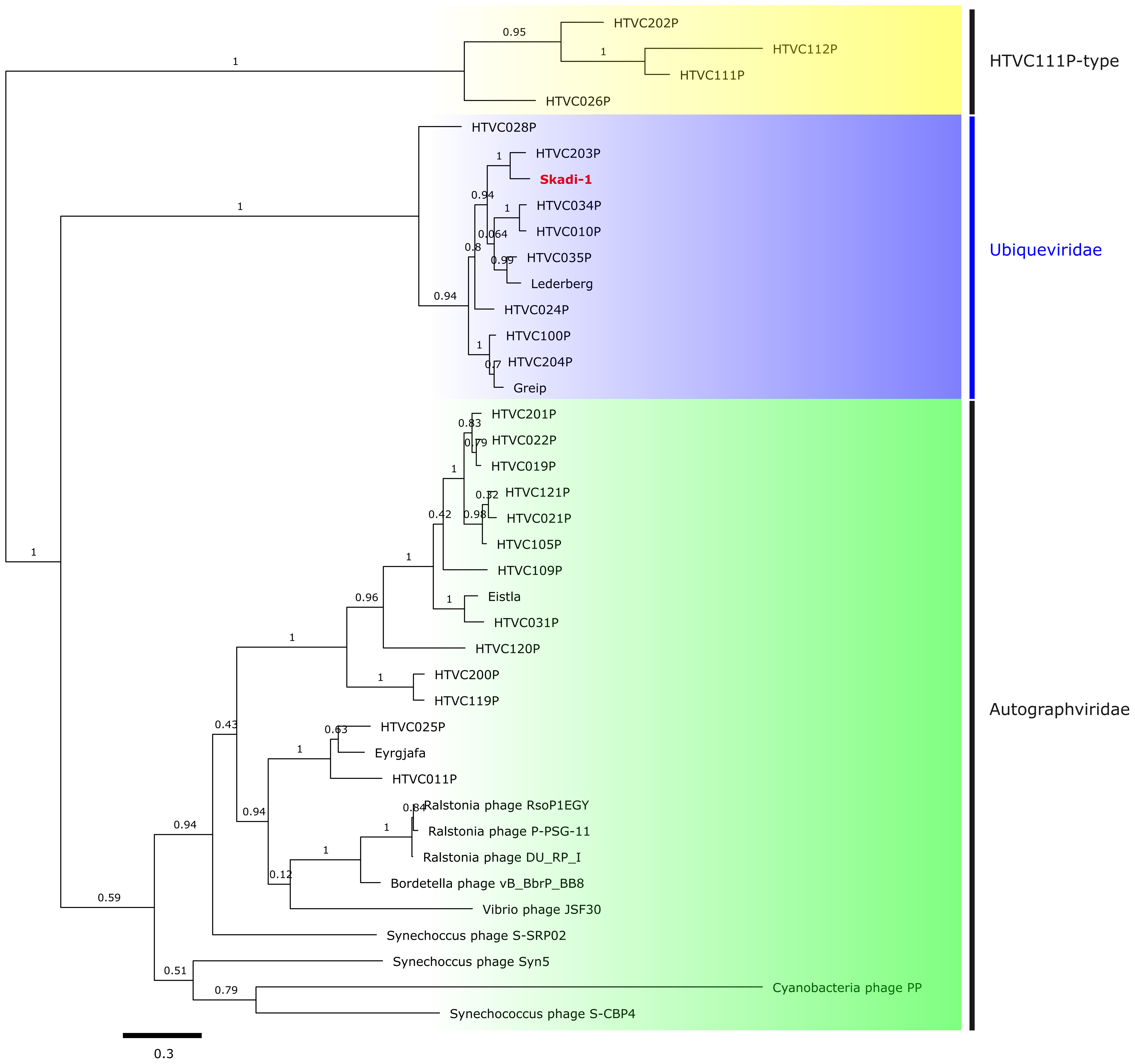

Supplementary Figure 2. Phylogeny of pelagiphage tail tube B genes. Unrooted neighbour-joining tree (100 bootstraps) of the tail genes related to Skadi-1's tail tube B protein found in Pelagibacter phages and representatives of other viral families. Branches are coloured to highlight the different taxonomic groups in relation to the proposed Ubiqueviridae. Pelagibacter phage genome types are marked by black bars.

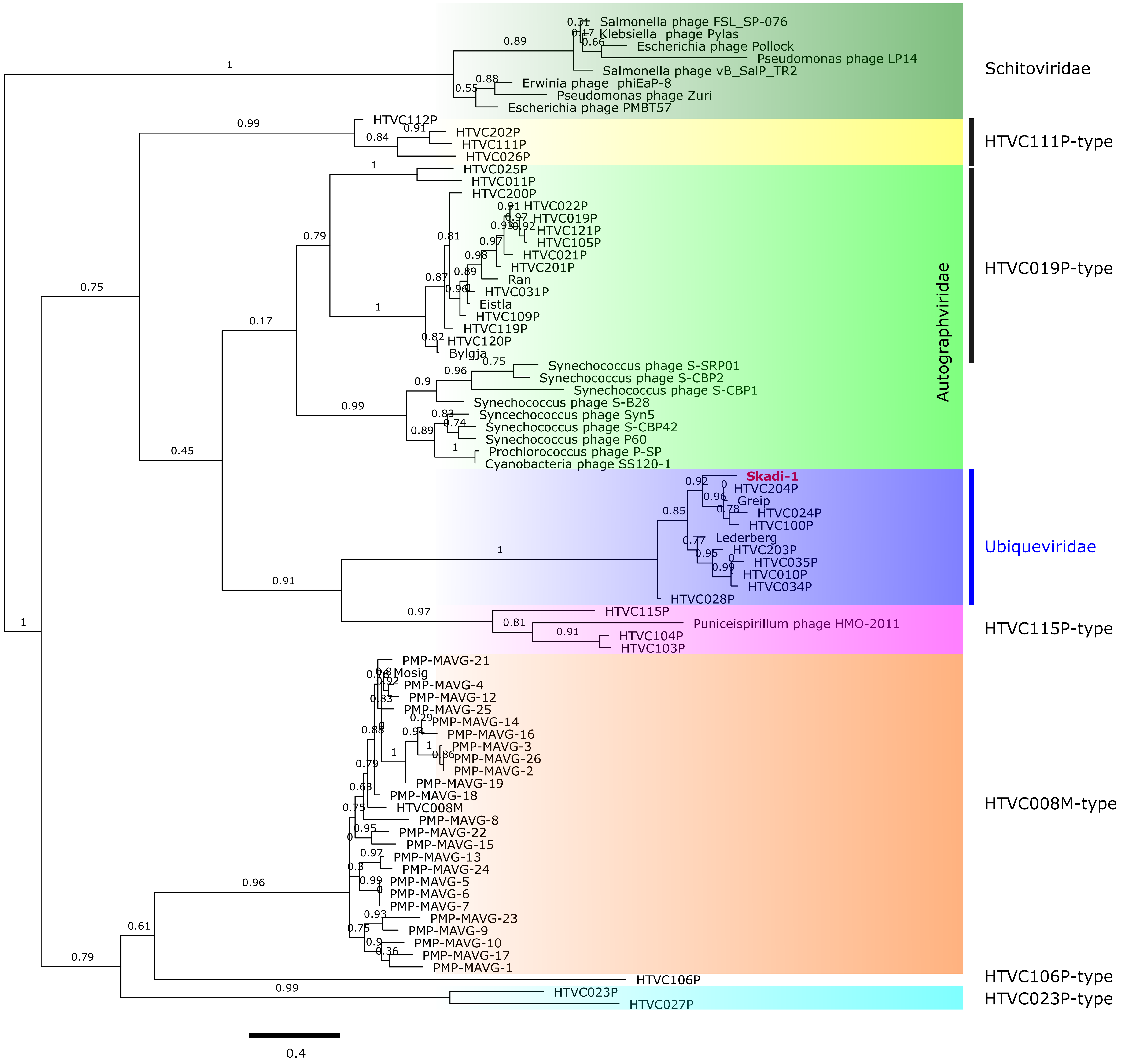

Supplementary Figure 3. Phylogeny of pelagiphage major capsid protein genes. Unrooted neighbour-joining tree (100 bootstraps) of the major capsid protein found in Skadi-1, Pelagibacter phages and representatives of other viral families. Branches are coloured to highlight the different taxonomic groups in relation to the proposed Ubiqueviridae. Pelagibacter phage genome types are marked by black bars.

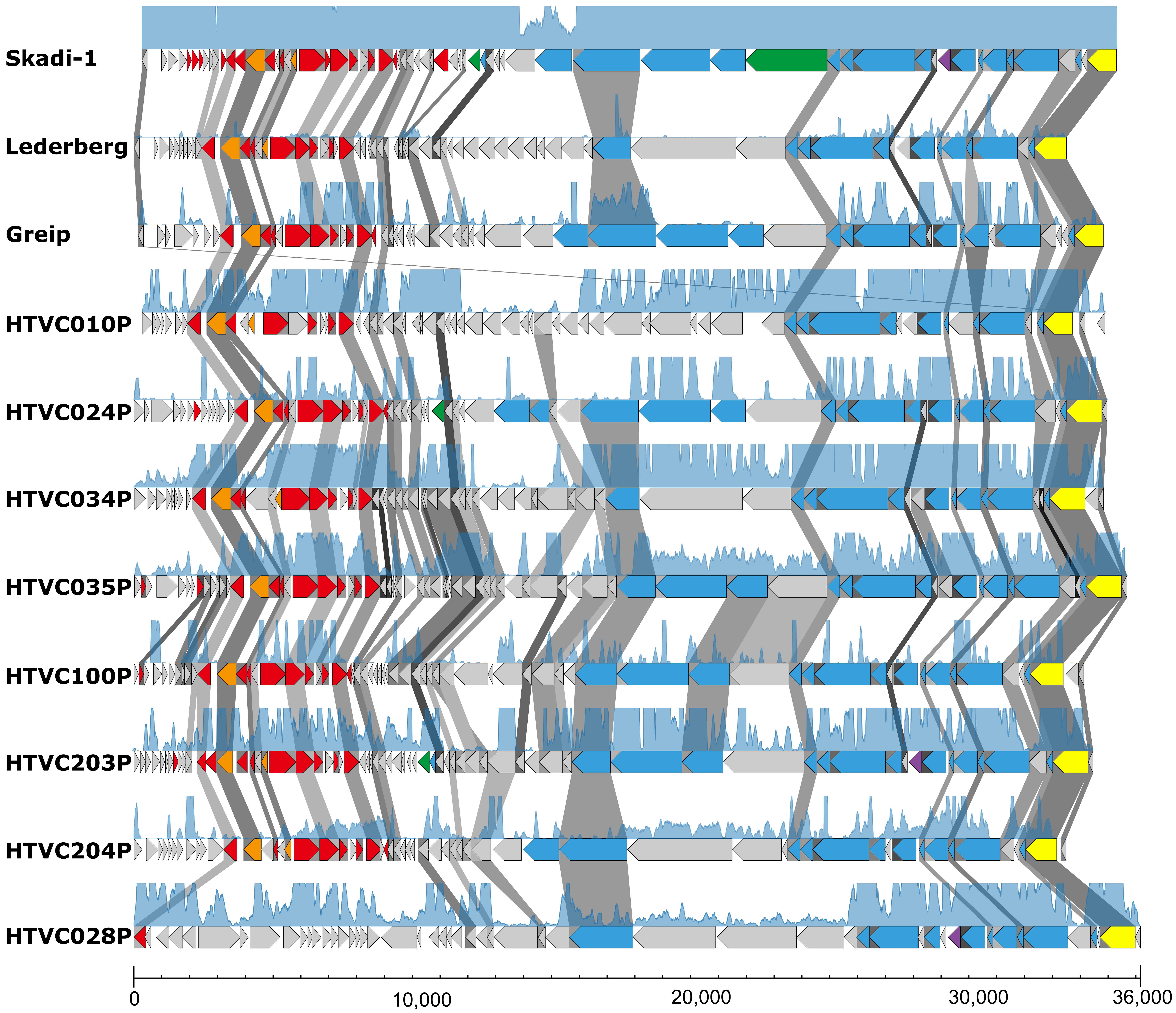

Supplementary Figure 4. Genomic map of all members of the proposed Ubiqueviridae family. Arrow directions represents coding strand (positive/negative), shading connecing the open reading frams (ORFs) indicate identity between shared genes. The filled blue line graph indicates per nucleotide coverage average (capped at 100) based on Global Ocean Virome (GOV2) reads mapped against phage genomes.

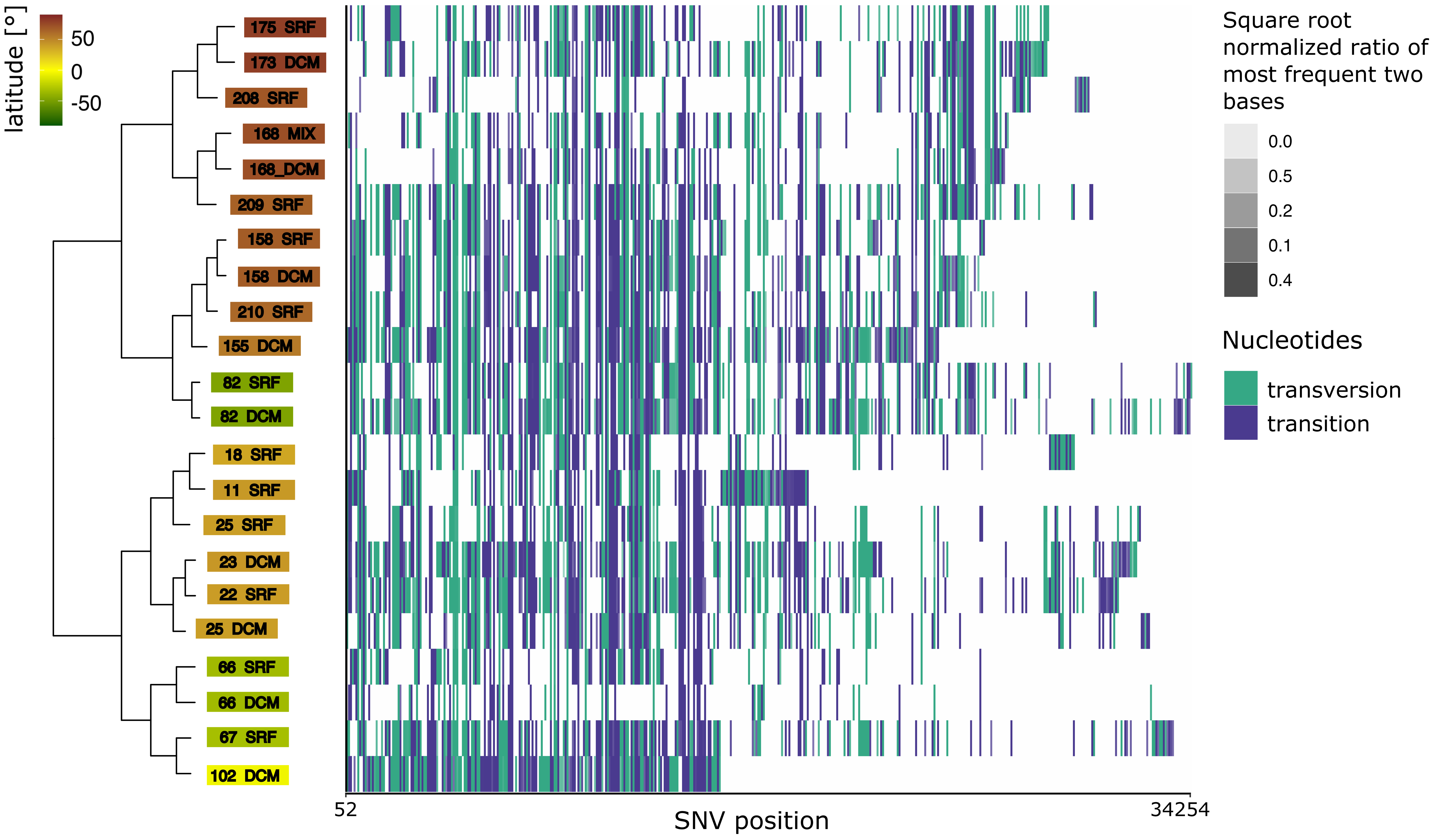

Supplementary Figure 5. Dendrogram representing the distances between Single Nucleotide Variant profiles of the reads from the GOV2 metagenomes mapped to the Skadi genome. A dendrogram representing the hierarchical clustering of the reads mapped to the Skadi genome is shown on the left side of the figure. Tips are color-coded based on the latitude where the sample was collected. A heatmap representing the profiles of the variable positions (461, columns) is shown on the right side matching the metagenome of the dendrogram tips. Nucleotide variants (compared to Skadi) are color-coded and shade of the cells represent the square-root normalized ratio of the two most frequent bases at the position (degree of variation in the position, lighter = more conserved- darker = more variable).

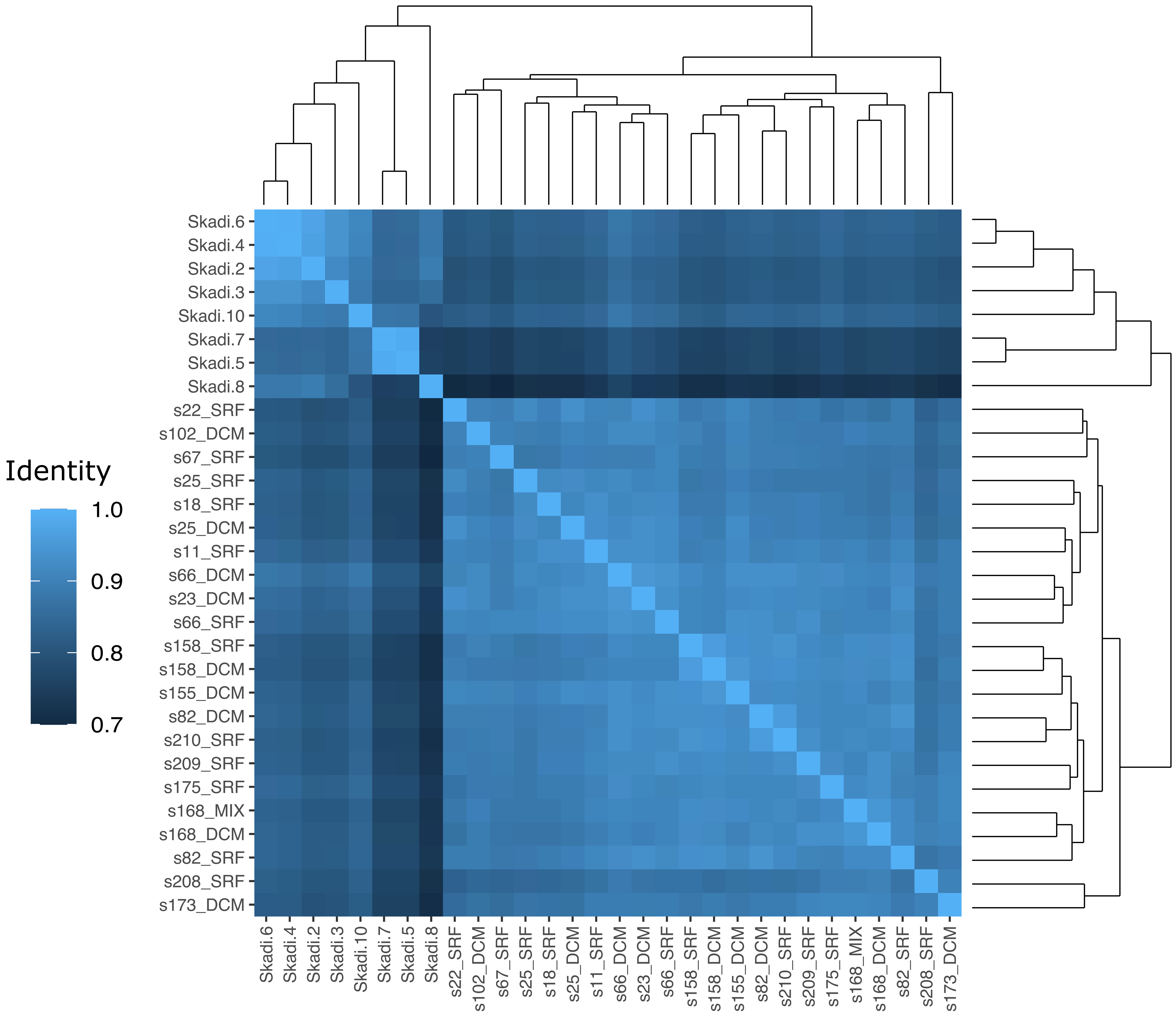

Supplementary Figure 6. WEC Skadi-like isolate genomes cluster together. Clustered heatmap of pairwise identity calculated between SNV positions using alignments of Skadi-like genomes against Skadi-1.

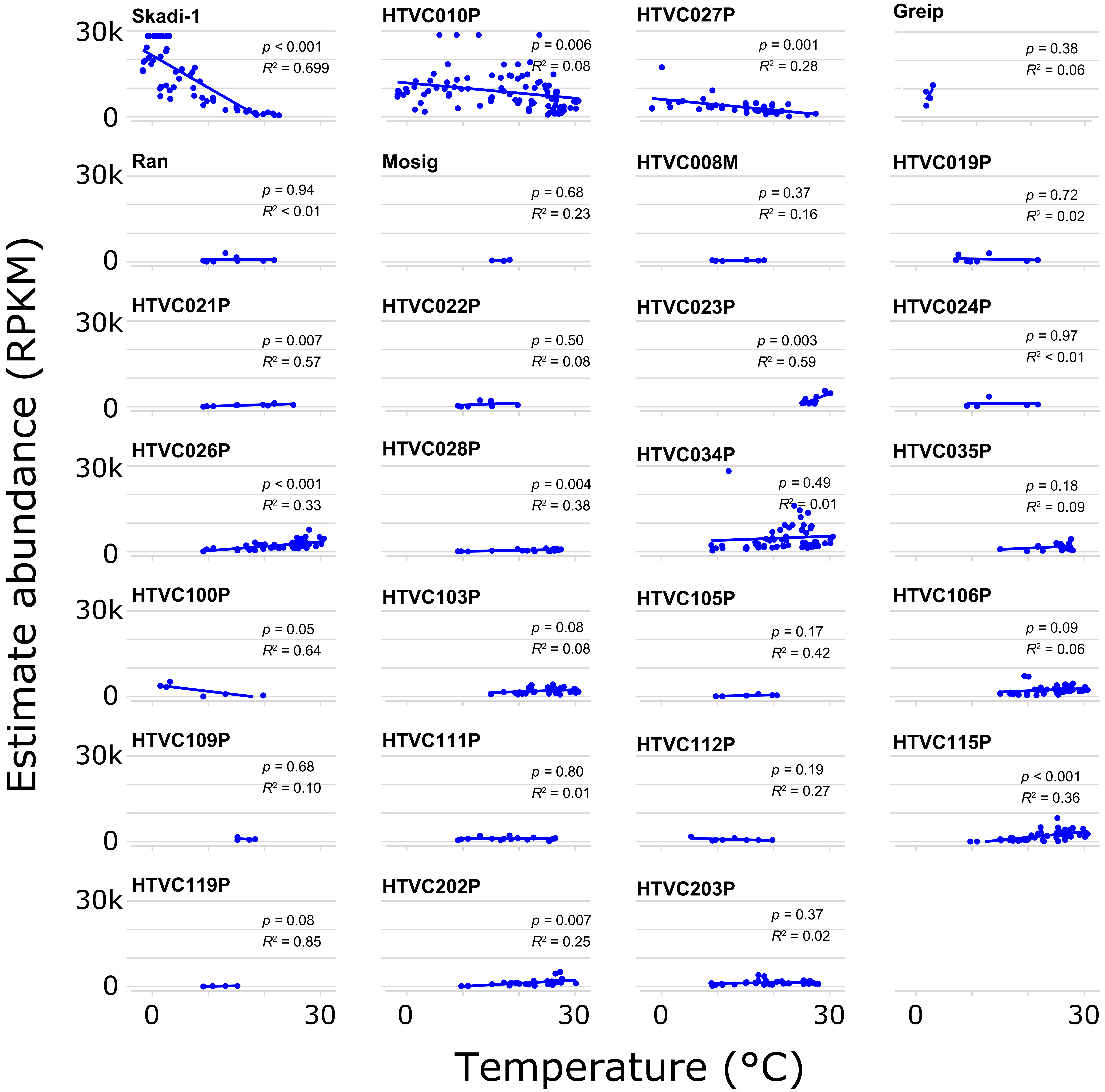

Supplementary Figure 7. Skadi-1 correlates with Latitude. Estimated abundances (RPKM) of isolated Pelagibacter phages from the GOV2 dataset with linear regression lines, samples with estimated abundances between zero and three RPKM in all viromes were excluded for clarity

Gene ID number of phage strain:

Core Gene Annotation	Greip	HTVC010P	HTVC024P	HTVC028P	HTVC034P	HTVC035P	HTVC100P	HTVC203P	HTVC204P	Lederberg	Skadi
**Tetratricopetide repeat**	8	15	48	37	50	55	40	54	45	10	50
**DNA binding**	9	14	49	38	51	56	51	55	46	11	51
**Acyltransferase**	10	13	50	40	52	57	52	56	47	12	52
**Tail tube B**	12	10	52	43	55	60	54	59	49	15	55
**Tail tube A**	13	9	53	44	56	61	55	60	50	16	56
**MCP**	14	8	54	45	57	62	56	61	51	17	57
**Tetratricopetide repeat**	15	7	55	46	58	63	57	62	52	18	58
**Portal**	16	6	56	47	59	64	58	63	53	19	59
**Hypothetical**	20	4	59	49	62	67	61	65	56	21	61
**TerL**	21	3	60	51	63	68	62	66	57	22	63

Supplementary Table 1. Shared core genes identified in each genome of the *Ubiqueviridae* .

Taxon / Group	morphotype	core genes	Species
Ubiqueviridae	podophage	10	11
Autographviridae	podophage	15	16
Kolga-type	siphophage	n.a.	1
Mosig-type	myophage	79	2
HTVC111P-type	podophage	28	4
HTVC103P-type	podophage	31	3
HTVC023P-tpye	podophage	33	2
HTVC106P-type	podophage	n.a.	1

Supplementary Table 2. Overview of shared core genes per pelagiphage type.
